# Sandfly Fever Viruses Attenuate the Type I Interferon Response by Targeting the Phosphorylation of JAK-STAT Components

**DOI:** 10.3389/fimmu.2022.865797

**Published:** 2022-06-01

**Authors:** Yarden Moalem, Yehonathan Malis, Konstantin Voloshin, Anna Dukhovny, Koret Hirschberg, Ella H. Sklan

**Affiliations:** ^1^ Department of Clinical Microbiology and Immunology, Sackler School of Medicine, Tel Aviv University, Tel Aviv, Israel; ^2^ Department of Pathology, Sackler School of Medicine, Tel Aviv University, Tel Aviv, Israel

**Keywords:** sandfly viruses, interferon, phleboviruses, STAT1, Jak1

## Abstract

Sandfly fever viruses are emerging Phleboviruses typically causing mild febrile illness. Some strains, however, can cause severe and occasionally fatal neuro-invasive disease. Like most viruses, *Phleboviruses* have devised various strategies to inhibit the type I interferon (IFN) response to support a productive infection. Still, most of the strategies identified so far focus on inhibiting the sensing arm of the IFN response. In contrast, the effect of sandfly virus infection on signaling from the IFN receptor is less characterized. Therefore, we tested the effect of sandfly fever virus Naples (SFNV) and Sicily (SFSV) infection on IFN signaling. We found that infection with either of these viruses inhibits signaling from the IFN receptor by inhibiting STAT1 phosphorylation and nuclear localization. We show that the viral nonstructural protein NSs mediates these effects, but only NSs from SFNV was found to interact with STAT1 directly. Thus, we tested the upstream IFN signaling components and found that Janus kinase 1 (Jak1) phosphorylation is also impaired by infection.

Furthermore, the NSs proteins from both viruses directly interacted with Jak1. Last, we show that IFN inhibition by SFNV and SFSV is most likely downstream of the IFN receptor at the Jak1 level. Overall, our results reveal the multiple strategies used by these related viruses to overcome host defenses.

## Introduction

Sandfly viruses cause self-limiting acute febrile disease. However, some viruses from this family can cause severe central and peripheral nervous system infections (1). Sandfly viruses belong to the genus *Phlebovirus* within the family *Phenuiviridae* of the *Bunyavirales* order. They are transovarially and horizontally transmitted by phlebotomine sandflies (2). Sandfly viruses have been known to affect naïve army troops traveling through endemic areas since the Napoleonic wars (3, 4). In addition, several sandfly virus outbreaks in non-military settings have been described over the years [see for some examples (5–8)]. Sandfly virus infections are endemic to several world regions, including Europe, the Mediterranean Basin, the Middle East, North Africa, and central Asia (9). A recent retrospective study showed that sandfly Toscana virus (TOSV) was the underlying cause of 4% of the meningoencephalitis case with an unknown cause in Southwest Germany (10). These viruses attract increasing attention due to their spread to new geographic regions and the ongoing isolation of new species. While these new species have unknown disease potential in humans, some show sequence identity to known disease-causing sandfly groups while others are lethal in mice (11–13).

Sandfly viruses contain a tri-segmented single strand-RNA genome (14). This genome encodes three structural proteins, including the nucleocapsid and two glycoproteins. The viral RNA polymerase is also present in the virion. In addition, sandfly viruses encode two nonstructural proteins, NSm and NSs, named after the RNA segment, they are encoded from. The nonstructural proteins are dispensable for viral replication. However, they were shown to significantly promote viral infection by inhibiting the antiviral type I interferon response (15).

Upon viral entry, host cell sensors recognize the incoming pathogen. After recognition, these proteins trigger a signaling pathway that leads to the production and secretion of type I IFN. The secreted IFN acts in both an autocrine and a paracrine manner, amplifying the antiviral response in the infected cell and preventing viral spread by activating the response in nearby cells (12). IFN binding to its receptor triggers the Jak-STAT pathway initiated by the phosphorylation and activation of Janus Kinase 1 (Jak1) and tyrosine kinase 2 (Tyk2) pre-associated with the IFN receptor, leading to the recruitment and phosphorylation of STAT1 and 2. In their phosphorylated state, STAT1 and STAT2 are activated and recruit the IFN-regulatory factor 9 (IRF9) to create the IFN-stimulated regulatory factor 3 (ISGF3) complex, which translocates into the nucleus. The ISGF3 complex then binds to IFN-stimulated response elements (ISRE) in the promotors of IFN-stimulated genes (ISG) and activates their expression. This pathway activates hundreds of ISGs, most of which have antiviral activities (16). Most studies focusing on sandfly NSs proteins showed inhibition of the sensing arm of the IFN response. TOSV was shown to employ a unique mechanism acting as ubiquitin ligase promoting proteasomal degradation of retinoic acid-inducible gene I (RIG-I), a cytosolic RNA sensor (17). Sandfly fever Sicilian virus (SFSV) was shown to inhibit interferon regulatory factor 3 (IRF3) binding with the IFN promoter (18). We hypothesized that, like related *Phlebovirus* that are not transmitted by sandflies (19–22), sandfly viruses might inhibit signaling from the interferon receptor as well. Thus, inhibiting the amplification of the response and the induction of an antiviral state in neighboring cells. Here we show that NSs inhibits IFN signaling from the IFN receptor by targeting Jak1 and provide further insights into this inhibition’s molecular details. Our results extend the knowledge regarding the strategies used by these emerging viruses to overcome the immune system.

## Materials and Methods

### Cell Culture, Viruses, Transfections, and Reagents

HEK293T cells stably expressing the ISG56 promoter (23), HEK293T, A549, U2OS, and Vero cells were cultured in Dulbecco’s modified Eagle’s medium (DMEM, Gibco) supplemented with 1% L-glutamine solution (Sartorius), 1% penicillin-streptomycin solution (Gibco) and 10% Fetal Bovine Serum (FBS, Gibco). HEK293-ISG56 medium supplemented with 400 µg/ml G418 (Millipore) and 1 µg/ml puromycin (*In vivo*Gen). All cell lines were maintained in a humidified incubator at 37°C with 5% CO2. Sandfly fever virus Naples (SFNV) and Sicily (SFSV) (Sabin strain) were a kind gift from the Israeli ministry of health’s central virology laboratory (Dr. Yaniv Lustig, Sheba Medical Center, Tel Hashomer, Israel). The virus stocks were propagated on Vero cells. Viral titers were determined by plaque assays on Vero cells. Polyethyleneimine (PEI, Polysciences) or Lipofectamine 3000 (Invitrogen) were used for plasmid DNA transfections of subconfluent cells. Interferon-alpha B2 was purchased from PBL (NJ; catalog no. 11115-1). Recombinant Human IL-28A/IFN-lambda 2 Protein was purchased from R&D systems (catalog no.1587-IL).

### Quantitative PCR

HEK293T cells were infected with sandfly viruses at a multiplicity of infection (MOI) of 1 or 0.1. The cells were treated with IFNα (200 U/ml) for 12 h. The cells were harvested 36 h post-infection. Total RNA was isolated using Tri reagent (Sigma). Purified RNA was reverse transcribed using iScript cDNA synthesis kit (Bio-rad). qPCR was performed using SYBR green (ABI) and the primers listed in **Table 1**. Relative mRNA levels were calculated by ΔΔCT method using Hypoxanthine Phosphoribosyltransferase 1 (HPRT) mRNA levels as a housekeeping control.

**Table 1:** List of primers for qPCR.

Primer name	Primer sequence (5’ to 3’)
OAS-F	CATCCGCCTAGTCAAGCACTG
OAS-R	CACCACCCAAGTTTCCTGTAG
MX1-F	CAGAGAGAAGGAGCTGGAAGAA
MX1-R	GCTGGCCTCCTGGTGATA
ISG15-F	CACCGTGTTCATGAATCTGC
ISG15-R	CTTTATTTCCGGCCCTTGAT
HPRT-F	TGACACTGGCAAAACAATGCA
HPRT-R	GGTCCTTTTCACCAGCAAGCT

### Antibodies

The following primary antibodies were used: Mouse anti-FLAG (Sigma, F3165, 1:1,000), Mouse anti-tubulin (Sigma, T9026, 1:10,000), Rabbit anti-STAT1 or 2 (Cell Signaling, 9127, 72604, 1:1,000), Rabbit anti-phospho-STAT1 or 2 (Cell Signaling, 88410, 9167, 1:1,000), Rabbit anti-V5 (Cell Signaling, 13202, 1:1,000), Rabbit anti-JAK1 (Santa-Cruz Biotechnology, sc-277; 1:400), Rabbit anti-phospho-JAK1 (Cell Signaling, 74129, 1:1000), Rabbit anti-lamin (Abcam, 1:1000). The secondary antibodies used were HRP anti-mouse and anti-rabbit (Jackson Immuno Research, 1:5000), Alexa fluor 488 goat anti-rabbit (Invitrogen, A11034, 1:500) or Alexa fluor 555 goat anti-rabbit (Invitrogen, A27039, 1:500).

### Western Blot

Cells were lysed with radioimmunoprecipitation (RIPA) buffer (50 mM Tris-HCl pH 7.4, 150 mM NaCl, 1% Triton x-100, 1% Sodium deoxycholate, 0.1% SDS, 1 mM EDTA) supplemented with a protease (Sigma) and phosphatase inhibitor cocktails (Roche PhosSTOP easy pack). The lysates were incubated for 30 min on ice and clarified by a 20 min centrifugation (20,000 g, 4°C). Following SDS–polyacrylamide gel electrophoresis, proteins were transferred to nitrocellulose membranes and blocked with 5% bovine serum albumin. Membranes were incubated with primary antibodies overnight, washed with tris-buffered saline (TBS) containing 0.001% Tween-20 (TBST), and incubated with the appropriate horseradish peroxidase-conjugated secondary antibody for 1 h followed by three TBST washes. Detection was performed using enhanced chemiluminescence.

### Subcellular Fractionation

A549 cells infected with SFSV or SFNV at an MOI of 1 were used for cellular fractionation experiments. The cells were stimulated with IFNα (1000 U/ml) for 30 min 24 h post-infection. For fractionation, the cells were washed with PBS supplemented with 5 mM EDTA. The cells were harvested in low salt buffer (10 mM HEPES pH 7.9, 10 mM KCl, 0.1 mM EDTA, 0.1 mM EGTA) supplemented with phosphatase and protease inhibitors and incubated on ice for 30 min. After 30 mins, 0.5% NP40 was added to the cells, and the cells were centrifuged at 4°C for 10 min at 10,000 g. The supernatants were kept as cytoplasmic fractions. The pellet was resuspended in high salt buffer (20 mM HEPES pH 7.9, 0.45 M NaCl, 1 mM EDTA, 1 mM EGTA) supplemented with phosphatase and protease inhibitors and incubated on ice for an additional 30 min. The cells were centrifuged at 4°C for 10 min at 14,000 g, and the supernatant was kept as a nuclear fraction. The cytoplasmic and nuclear fractions were analyzed by Western blot with a pSTAT1 antibody. Tubulin was used as a cytosolic marker, and lamin was used as a nuclear marker.

### Immunofluorescence

A549 or Vero cells were grown on coverslips 24 h following infection or transfection. The cells were stimulated with IFNα (1,000 U\ml) for 30 min and fixed with 4% paraformaldehyde for 20 min. After three PBS washes, the fixed cells were permeabilized using ice-cold 100% methanol for 10 min, followed by three additional PBS washes. Blocking was with 5% FBS and 0.3% Triton X-100 in PBS for 1 h. Cells were then incubated at room temperature for 1 h or overnight with the primary antibodies, and after three PBS washes were incubated for an additional hour with the secondary antibody. Cell nuclei were stained with 4-6′ diamidino-2 phenylindole (DAPI, Sigma). Cells were visualized using a confocal laser microscope (LSM800, Carl Zeiss).

### Plasmids

Sandfly NSs plasmids were cloned by amplifying the NSs sequences from total RNA produced from SFNV or SFSV infected Vero cells. The sequences were reverse transcribed and cloned into XhoI and XbaI sites of a modified pEF-IRES-puro vector encoding a C-terminal or N-terminal 3xFLAG tags. pTWIST-CMV expressing SFNV NSs with an N-terminal V5-tag was purchased from Twist Bioscience. pTWIST-CMV expressing Jak1 (accession # NP_001307852) with a C-terminal V5-tag was purchased from Twist Bioscience. pLV-WT-STAT1 and pLV-STAT2 were a gift from George Stark (Addgene plasmid # 71454 and 71451). pCAGGS-VP35 containing FLAG-tagged Sudan Ebola virus VP35 protein was a gift from Prof. Leslie Lobel (Ben-Gurion University, Israel). pcDNA-Wnt7A-V5 was a gift from Marian Waterman (Addgene plasmid # 35933). The plasmid expressing suppressor of cytokine signaling 3 (SOCS3) pCMV SOCS-3 was a gift from Ronald Kahn (Addgene plasmid # 11486).

### Luciferase Assay

HEK293T cells stably expressing the ISG56 promoter were cultured in 24-well plates and cotransfected with 500 ng pIRES-3xFLAG-NSs, pcDNA-NS4A-3×FLAG or empty pIRES-3×FLAG plasmids and 10 ng Renilla SV40 plasmid using PEI. Following transfection (24 h), the cells were stimulated with human IFNα (1,000 U/ml) for an additional 12 h. The cells were lysed with luciferase reporter lysis buffer (Promega), and luciferase activity was determined using a GloMax^®^ 96 microplate luminometer (Promega). Renilla luciferase values were used as a transfection control.

### Immunoprecipitation

HEK293T cells cotransfected with the indicated plasmids were lysed using lysis buffer (150 mM sodium chloride, 1% Triton X-100, 50 mM Tris pH 8, 1 mM sodium orthovanadate) supplemented with protease (Sigma) and phosphatase inhibitor cocktails (Roche PhosSTOP easy pack). The cells were incubated with lysis buffer on ice. The lysates were then clarified by centrifugation (15 min, 18,800 g, 4˚C). Protein lysates were precleared with protein A\G plus (Santa Cruz Biotechnology). Rabbit anti-V5 antibodies (1:200) or M2 affinity gel beads (Sigma) were added for overnight incubation. The next day A\G beads were added for 1.5 h at 4˚C. The samples were then washed three times using lysis buffer. The samples were eluted by 5 min boiling in sample buffer and analyzed by western blot.

### Generation of STAT1 Knockout Cells

The lentiviral vector pXPR-STAT1 containing sgRNA targeting STAT1 (GAGGTCATGAAAACGGATGG) and hSpCas9 was previously described (24), a gift from Prof. Eran Bacharach). Cells expressing a non-targeting sgRNA were used as control. Lentiviral particles containing these pXPR lentivectors were prepared as described above and used for infection of HEK293T cells. Clones were selected with puromycin (0.6 µg\ml). Individual colonies were expanded and tested for STAT1 expression using immunoblotting.

## Results

### Infection With Sandfly Fever Virus Naples and Sicily Inhibits IFN-Induced ISG Expression

**Figure 1:**
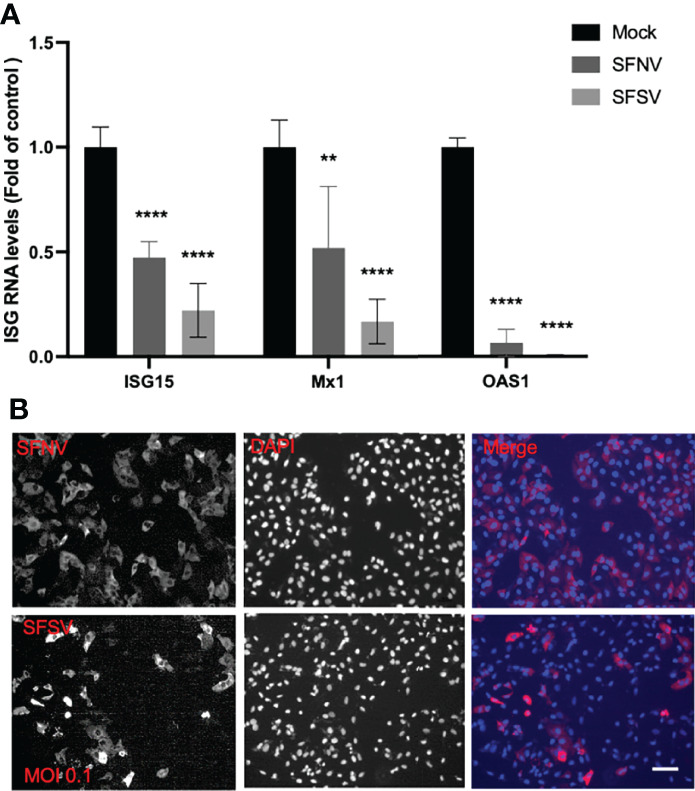
Sandfly virus infection inhibits type I IFN signaling. **(A)** HEK293T cells were infected with SFNV at an MOI of 1, and SFSV at an MOI of 0.1. The next day the cells were treated with IFNα (200 U/ml) for 12 h. The expression of ISGs (ISG15, Mx1, and OAS1) was quantified using qPCR. Shown are representative results from 2 independent experiments performed in triplicates (**P<0.01, ****P<0.0001, one-way ANOVA, Dunnett’s multiple comparisons test). **(B)** A549 cells were infected with SFNV or SFSV as described above. The next day, the cells were fixed and stained with anti-SFNV or SFSV specific antibodies. Bar=500 µM.

Activation of type I IFN signaling results in the expression of ISGs from promoters containing interferon-sensitive response elements [ISRE, (16)]. To investigate whether sandfly viruses can inhibit IFN-signaling, we tested the induction of several ISGs following infection with sandfly fever virus Naples (SFNV) or Sicily (SFSV) and stimulation with IFN, including interferon-stimulated gene 15 (ISG15), myxovirus-resistance A (Mx1), and oligoadenylate synthetase 1 (OAS1, **Figure 1A**). Infection levels were confirmed in A549 cells using immunofluorescence with sandfly virus-specific antibodies (**Figure 1B**). In both SFNV and SFSV infected cells, the activation of all examined ISGs was suppressed. The highest levels of inhibition compared to the mock-infected IFN stimulated cells were observed with OAS1, 282.6-folds with SFSV, and only 15.5-folds with SFNV. ISG15 levels dropped by 2.12-folds in SFNV infected cells and 4.5-folds in SFSV infected cells. A decrease of 2-folds in SFNV and 6-folds in SFSV was observed with Mx1. These results indicate that sandfly infection inhibited IFN-induced ISG expression.

### Sandfly Viruses Inhibit the Phosphorylation of STAT1 and 2

**Figure 2:**
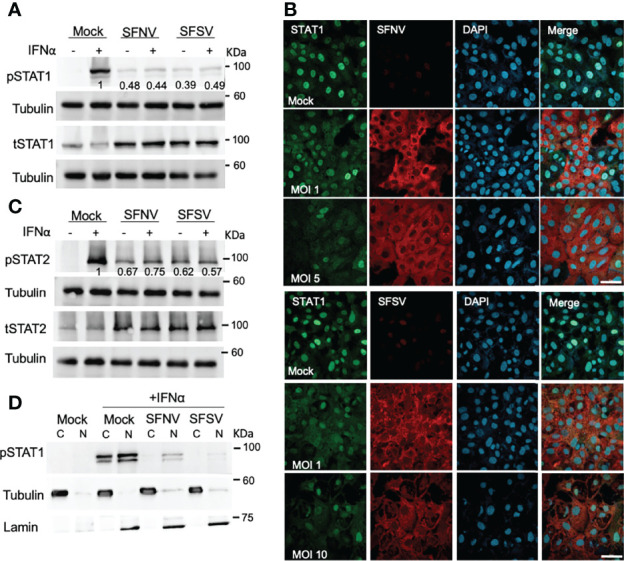
Sandfly virus infection decreases the phosphorylation of STAT1 and 2. **(A)** SFNV, and SFSV inhibits STAT1 phosphorylation. A549 cells were infected with SFSV or SFNV at an MOI of 1. The cells were stimulated with IFNα (1000 U/ml) for 30 min 24 h post-infection. The lysates were analyzed by western blot using specific antibodies for phosphorylated or total STAT1. Tubulin was used as a loading control. Numbers below the bands represent average bands intensity from three independent experiments quantified using ImageJ and are expressed as a percent of mock-infected and stimulated control. **(B)** SFNV and SFSV affect STAT1 localization. Vero cells were infected with SFNV at the indicated MOIs (left). The next day, cells were treated with IFNα (1000 U/ml) for 30 min, then fixed and co-stained with anti-Naples (red) and anti-STAT1 (green) specific antibodies. DAPI was used for nuclei labeling (blue). A similar experiment was performed following SFSV infection. Staining was with anti-Sicily antibodies (right). Scale bar=50µM. **(C)** SFNV and SFSV infection inhibit STAT2 phosphorylation. Cells were infected and stimulated and analyzed as described above for STAT1. Analysis was performed using STAT2 and phospho-STAT2 specific antibodies. **(D)** Subcellular fractionation of Infected cells. A549 cells were infected with SFSV or SFNV at an MOI of 1. The cells were stimulated with IFNα (1000 U/ml) for 30 min 24 h post-infection. The cells were fractionated, and the lysates were analyzed by western blot using specific antibodies for phosphorylated STAT1. Tubulin was used as a cytoplasmic marker, and lamin was used as a nuclear marker.

STAT activation is a critical event in IFN-signaling and thus is targeted by many viral proteins (16). To test whether sandfly virus infection affects STAT phosphorylation, we analyzed phosphorylated STAT (pSTAT) levels in SFNV or SFSV infected cells stimulated with IFN. Infection with both viruses considerably decreased pSTAT1 levels (**Figure 2A**). In the infected cells, pSTAT1 levels dropped to 44% in SFNV and 49% in SFSV compared to mock-infected IFN stimulated cells. In contrast, total STAT1 levels did not decrease following infection and stimulation, suggesting that SFNV and SFSV affect STAT1 phosphorylation and not STAT1 stability (**Figures 2A, B**). Total STAT levels were, in fact, higher following sandfly infection. This could be explained by the fact that STAT is an ISG and can be induced during the 24-hour long infection (25). To confirm these results, the effect of sandfly virus infection on the STAT1 localization was further examined. SFNV or SFSV infected cells were stimulated with IFNα, and STAT1 localization was determined using immunofluorescence. As shown in **Figure 2B**, STAT1 is mainly localized at the nucleus in control stimulated cells. SFNV infection decreased STAT1 nuclear staining. Similar results were observed in SFSV infected cells. In addition, STAT1 nuclear staining decreased with increased MOI. These results support the observed decrease in STAT1 phosphorylation in infected cells. To test if SFNV and SFSV infection affect STAT2, its phosphorylation levels were also tested in infected cells (**Figure 2C**). The results were similar to those observed with STAT1, i.e., pSTAT2 levels dropped to 75% in SFNV and 57% in SFSVof their levels in the mock-infected controls despite the IFN stimulation. Total STAT2 levels were higher following sandfly infection, as with STAT1. The localization of pSTAT1 in infected cells was further confirmed by subcellular fractionation (**Figure 2D**). pSTAT1 was eliminated from the cytoplasm and significantly reduced in the nuclei of the infected cells. These results suggest that the sandfly virus infection affects STAT1 and 2 phosphorylation.

### Sandfly Virus NSs Expression Attenuates IFN-Signaling

**Figure 3:**
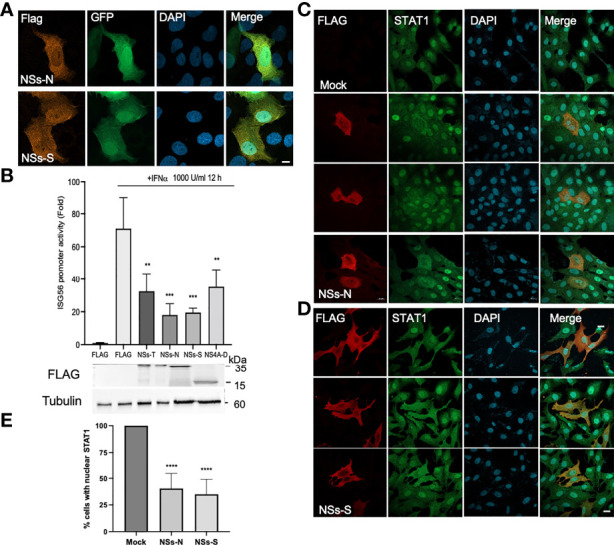
NSs inhibits IFN mediated transcription and affects STAT1 localization. **(A)** Intracellular localization of NSs-N and NSs-S proteins. FLAG-tagged NSs proteins were cotransfected into U2OS cells with pEGFP-N1. The cells were fixed and stained with anti-FLAG antibodies. **(B)** NSs mediated inhibition of IFN mediated transcription. ISG56 luciferase reporter cells were transfected with plasmids expressing sandfly NSs, Dengue NS4A, or an empty plasmid. A Renilla luciferase-expressing plasmid was used as a transfection control. After 24 h, the cells were treated with IFNα (1000 U\ml). The cells were harvested after an additional 12 h, and luciferase activity was determined. Shown is a representative experiment from three independent experiments showing similar results (**P<0.01, ***P<0.001, One way ANOVA, Dunnett’s multiple comparisons test). The lysates from the luciferase assay were subjected to western blot analysis using FLAG antibodies. Tubulin was used as a loading control. **(C, D)** NSs affects STAT1 localization. Vero cells were transfected with FLAG-tagged NSs expressing plasmids. The cells were stimulated with IFNα (1000 U/ml) for 30 min 24 h post-transfection, then fixed and stained with anti-FLAG (red) and anti-STAT1 (green) specific antibodies. DAPI was used for nuclei labeling (blue). **(E)** The percentage of transfected cells with nuclear STAT1. Results are representative of three independent experiments (n=50 cells for each condition ****P<0.0001, One way ANOVA, Dunnett’s multiple comparisons test). Scale bar = 10 µM.

TOSVs lacking NSs could not block IFN production (26). Thus, we tested if NSs is responsible for abrogating IFN-signaling. First, FLAG-tagged NSs proteins were produced, and their intracellular localization was determined (**Figure 3A**). Both proteins localized at the host cell’s cytoplasm colocalizing with GFP. In contrast to SFSV NSs (NSs-S), SFNV NSs (NSs-N) were excluded from the nucleus of most cells (not shown). The effect of NSs from TOSV (NSs-T), NSs-N, and NSs-S on the transcription levels of an IFN responsive promoter was further determined using an ISG56 luciferase reporter (23). Dengue virus NS4A (NS4A-D), known to inhibit IFN signaling, was used as a positive control (27). IFNα stimulation resulted in robust induction of the ISG promotor compared to the unstimulated control (71-folds, **Figure 3B**). However, promoter activity was significantly reduced by 55% in cells expressing NSs-T, 74% in cells expressing NSs-N, and 72% in cells expressing NSs-S. A reduction of 50% was observed with NS4A-D. The correct expression of the viral proteins was confirmed by western blot. These results indicate that the sandfly NSs proteins can inhibit IFN signaling. To reveal this inhibition’s mechanistic details, we tested if NSs can affect STAT1 localization following IFNα stimulation. In mock-transfected and stimulated cells STAT1 is mainly detected in the nucleus (**Figures 3C–E**). STAT1 did not label the nucleus of 59.6 ± 14.1% of NSs-N expressing cells and 64.9 ± 14% of NSs-S expressing cells, suggesting that NSs expression decreases its nuclear accumulation.

**Figure 4:**
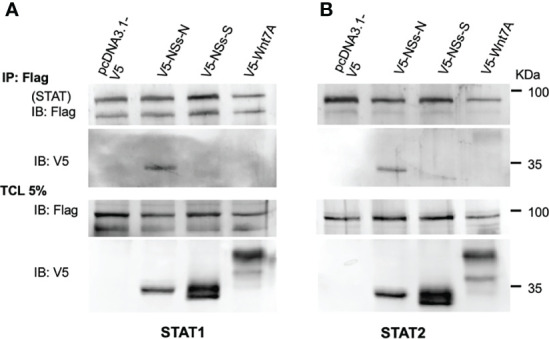
NSs-N interacts with both STAT1 and STAT2, but NSs-S does not. **(A)** HEK293 cells were cotransfected with FLAG-tagged STAT1 or STAT 2 **(B)** expressing plasmids and V5-tagged sandfly NSs expressing plasmids. An empty plasmid and a plasmid expressing an unrelated protein V5-Wnt7A were used as controls. Immunoprecipitation (IP) was performed 48 h post-transfection using FLAG beads. Total cell lysates (TCL) and IP products were analyzed by western blot using the indicated antibodies.

To further understand how NSs inhibits STAT activity, we tested if NSs interacts with these proteins. Immunoprecipitation experiments were performed in cells overexpressing STATs and NSs. Interestingly, only NSs-N was pulled down by both STAT1 and STAT2 (**Figures 4A, B**). In contrast, NSs-S and Wnt7A, an unrelated protein used as a negative control, did not interact with the STAT proteins. These results suggest that NSs have a role in sandfly virus-mediated inhibition of IFN signaling through distinct mechanisms.

### NSs Interacts With Jak1 and Affects Its Phosphorylation

**Figure 5:**
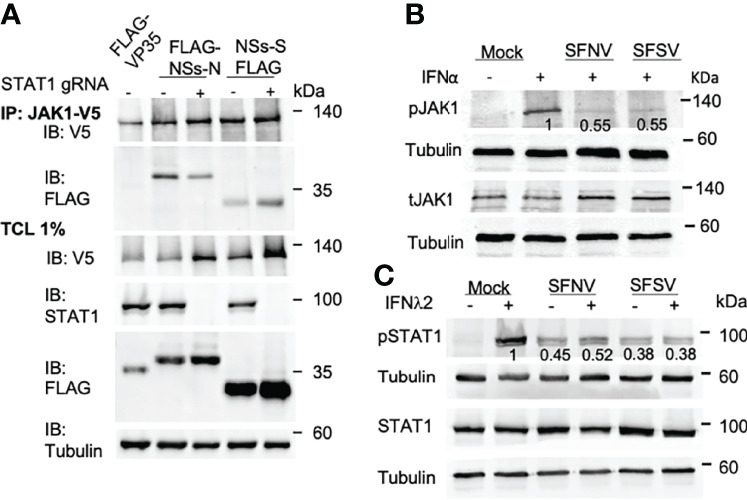
NSs interact with Jak1 independently and inhibits its phosphorylation. **(A)** HEK293 cells expressing NT sgRNA or sgRNA for STAT1 were cotransfected with plasmids expressing V5-Jak1 and FLAG-tagged NSs. IP was performed 24 h post-transfection using an anti-V5 antibody. Total cell lysates (TCL) and IP products were analyzed by western blot using the indicated antibodies. **(B)** SFNV and SFSV infection inhibits Jak1 phosphorylation. A549 cells were infected with SFNV or SFSV. The next day the cells were treated with IFNα (1000 U\ml) for 30 mins. Western blot analysis was performed as described above using Jak1 and phospho-Jak1 specific antibodies. Tubulin was used as a loading control. Numbers below the bands represent average bands intensity from three independent experiments quantified using ImageJ and are expressed as a percent of mock-infected and stimulated control. **(C)** SFNV and SFSV infection inhibits IFNλ2 signaling. A549 cells were infected with SFNV or SFSV. The next day the cells were stimulated with IFNλ2 (70 ng\ml) for 20 mins. Western blot analysis was performed using total STAT1 and phospho- STAT1specific antibodies. Tubulin was used as a loading control.

NSs-S did not interact with STAT1 and STAT2. Thus, we hypothesized that its effect on STAT phosphorylation stems from an inhibition of an upstream step. To test this possibility, the ability of NSs to interact with Jak1 was also determined (**Figure 5A**). After its activation, Jak1 binds and phosphorylates STATs (15). Thus, to confirm that the putative NSs interaction with Jak1 is direct, immunoprecipitation was also performed in CRISPR mediated STAT1 knockout cells.

Surprisingly, both NSs-N and NSs-S interacted and Jak1. In contrast, such an interaction was not observed with the Ebola virus VP35, used as a negative control. NSs from both viruses interacted with Jak1 in STAT1 knockout cells, suggesting that this interaction is direct and independent of STAT1 expression.

To test if the interaction of NSs with Jak1 has functional consequences on Jak1 itself, we tested the phosphorylation levels of Jak1 in IFN stimulated SFNV and SFSV infected cells (pJak1, **Figure 5B**). pJak1 levels decreased to 55% in SFNV and SFSV infected cells, compared to mock infected stimulated cells. Total Jak1 levels, however, did not decrease. These results indicate that SFNV and SFSV have a significant effect on the phosphorylation levels of Jak1. To test if the observed effect is downstream of the IFN receptor, we stimulated SFNV and SFSV infected cells with IFNλ2, a type III IFN, which binds to different IFN receptors (16). SFNV and SFSV infection inhibited STAT phosphorylation in IFNλ2 stimulated cells, indicating that the inhibition is most likely downstream of the receptor at the Jak1 level (**Figure 5C**). Taken together, our results indicate that sandfly NSs inhibits IFN signaling downstream of the receptor at the Jak1 level.

## Discussion

For a productive infection to occur, viruses have to overcome the host’s first line of defense, the type I IFN response. In this study, we asked if, in addition to various approaches used by *Phleboviruses* to inhibit mostly viral sensing [summarized in (15)], these viruses also utilize additional tactics to inhibit signaling from the IFN receptor. We found that infection with SFNV and SFSV inhibits STAT1 and 2 phosphorylation. NSs proteins from both viruses were found to mediate this inhibition. However, only NSs from SFNV interacted with the STAT proteins. To confirm that the inhibition is not at the receptor level, we stimulated cells with IFNλ2, a type III IFN that uses the same signaling pathway following the stimulation of a different receptor complex (16). IFNλ2 stimulated infected cells were unable to transduce the IFN signal indicating that the inhibition is most likely downstream of the receptor. The binding of type I or type III IFNs to their receptors triggers the phosphorylation of Jak1 and Tyk2, which in turn phosphorylate the receptors, causing the recruitment and phosphorylation of STAT proteins (16). We found that IFN signaling is inhibited downstream of the receptor and upstream of the STAT proteins in infected cells. Thus, this inhibition most likely occurs at the Jak1 and/or Tyk2 levels. Indeed, our results indicate that Jak1 interacts with the NSs proteins from both viruses and that Jak1 phosphorylation is inhibited in stimulated cells upon infection. Inhibition of Tyk2 as well cannot be ruled out at this stage. Inhibition of Jak1 phosphorylation is a known strategy previously employed by several viruses, including, for example, Marburg virus, Langat virus, West-Nile virus, Measles virus, HSV-1, and others to inhibit IFN signaling (28–32).

The viral NSs protein was previously implicated in inhibiting IFN production following infection with sandfly or other *bunyaviruses* (18, 26, 33–39). In our luciferase reporter assay, NSs mediated inhibition levels of transcription from an IFN responsive promoter were comparable to NS4A, a known IFN inhibitor (27). However, upon overexpression, NSs could only decrease STAT1 nuclear localization in ~60% of the transfected cells. These results could stem from different expression levels of NSs in different NSs expressing cells and other factors. However, the involvement of an additional viral protein that could affect IFN signaling during infection could not be ruled out in this stage. A recent study used a different experimental approach and tested IFN and ISG induction in non-stimulated SFSV infected cells (40). This study concluded that SFSV cannot inhibit IFN signaling and does not bind the STAT proteins. Thus, our results are only partially in agreement. One possible explanation for this discrepancy could be that SFSV could promote an early IFN-independent induction of a subset of ISGs as previously described for several viruses (41, 42). Despite their relatively high NSs sequence identity (54%), NSs-N does not inhibit viral sensing like NSs-T. Thus SFNV will most likely need to obtain other means to inhibit IFN signaling to produce productive infections (43). In contrast, NSs-N and NSs-S share approximately 20% sequence identity and, according to our results, have some similarities in inhibiting IFN signaling, i.e., inhibition of Jak1 phosphorylation. It is still unclear why NSs-N interacts with the STAT proteins to achieve this inhibition while NSs-S does not. Interestingly, severe fever with thrombocytopenia syndrome (SFTS) NSs shares a similar 20% sequence homology with NSs-N, uses an entirely different strategy to disrupt IFN-signaling by NSs mediated hijacking of STAT1 and 2 into inclusion bodies (44).

Two main mechanisms might be envisioned for the inhibition of Jak1 phosphorylation by these viruses; a steric inhibition preventing its interaction with essential kinases and recruitment of phosphatases which abolish the phosphorylation. A steric inhibition can be caused by the NSs itself or by other proteins recruited by NSs. For example, ubiquitin-specific peptidase 18 (USP18) inhibits the activity of Jak1 by blocking its interaction IFN receptor (45). Although we showed that the inhibition of Jak1 phosphorylation occurs downstream of the receptor, USP18 might still be able to interact with Jak1 and both type I and III IFN receptors. Another candidate might be the suppressor of cytokine signaling 3 (SOCS3), which binds and directly inhibits the catalytic domains of Jak1 and Tyk2 (46). However, preliminary experiments in our laboratory (not shown) indicate that NSs proteins from both SFNV and SFSV do not interact with SOCS3. Thus, the exact details or accessory proteins used by sandfly proteins to inhibit IFN signaling should be further determined. Our results indicate that SFNV and SFSV inhibit IFN signaling and highlight the diverse strategies used by *Phleboviruses* to inhibit IFN production. A better understanding of NSs mediated inhibition of IFN-signaling might provide insights into disease pathogenesis and might assist in developing means to prevent the associated diseases.

## Data Availability Statement

The original contributions presented in the study are included in the article/supplementary material. Further inquiries can be directed to the corresponding author.

## Author Contributions

ES conceived and designed the research and wrote the paper. YMO designed and performed the experiments, analyzed the data, and revised the manuscript. YMO, KV, and AD performed some of the experiments. KH analyzed data. All authors contributed to the article and approved the submitted version.

## Funding

This study was funded in part by a research grant awarded by the Tel Aviv University Center for Combatting Pandemics.

## Conflict of Interest

The authors declare that the research was conducted in the absence of any commercial or financial relationships that could be construed as a potential conflict of interest.

## Publisher’s Note

All claims expressed in this article are solely those of the authors and do not necessarily represent those of their affiliated organizations, or those of the publisher, the editors and the reviewers. Any product that may be evaluated in this article, or claim that may be made by its manufacturer, is not guaranteed or endorsed by the publisher.
